# The mechanism of nicotinamide on reducing acute lung injury by inhibiting MAPK and NF-κB signal pathway

**DOI:** 10.1186/s10020-021-00376-2

**Published:** 2021-09-20

**Authors:** Qun Zhang, Junyao Li, Haixia Zhong, Yanling Xu

**Affiliations:** 1 Department of Respiratory and Critical Care Medicine, The Second Hospital of Jilin University, No. 218, Ziqiang Street, Nanguan District, Changchun, 130000 Jilin China; 2 Department of Respiration, General Hospital of Jilin Tonghua Mining Co., Ltd, No. 67, Nanling Street, Hunjiang District, Baishan, 134300 Jilin China

**Keywords:** Acute lung injury, Nicotinamide, MAPK, AKT/NF-κB

## Abstract

**Background:**

Acute lung injury is an important factor that leads to the death of patients with pneumonia. Previous studies have shown that nicotinamide (NAM) plays a role in reducing cell damage, so this study explored the mechanism by which NAM functions in acute lung injury.

**Methods:**

We explored the mechanism by which NAM affects acute lung injury in vivo and in vitro by qRT-PCR, western blotting and ELISA.

**Results:**

The results showed that NAM could significantly reduce lung injury and proinflammatory mediator accumulation. Further mechanistic studies showed that NAM could significantly inhibit the MAPK and AKT/NF-κB signaling pathways.

**Conclusion:**

These results suggested that NAM may reduce the release of proinflammatory mediators by inhibiting the MAPK and AKT/NF-κB signaling pathways and ultimately alleviate lung injury.

**Supplementary Information:**

The online version contains supplementary material available at 10.1186/s10020-021-00376-2.

## Introduction

Pneumonia is a respiratory disease that is commonly observed in the clinic, and it can be triggered by many causes (Bedeley et al. 2021). At present, the most common types of pneumonia are bacterial pneumonia and viral pneumonia (Yu and Fei 2016). Studies have shown that both bacterial pneumonia and viral pneumonia can lead to acute lung injury (Kumar 2020). COVID-19, which is spreading all over the world, also causes acute lung injury (Kumar 2020). Severe acute lung injury can even lead to death of patients. The mainstream approaches for treating pneumonia in the clinic are antibacterial or antiviral drugs (Liapikou et al. 2019). These antibacterial or antiviral drugs can significantly kill or inhibit the proliferation of viruses or bacteria (Kollef and Betthauser 2019; Vachharajani and McCall 2020). However, many antibiotics can cause serious side effects, and antibiotics can also cause bacteria to release a large number of endotoxins and other proinflammatory substances, aggravate lung inflammation, and even lead to cytokine storms (Cunha 2001; Wong et al. 2016). Enhancing the body's immunity also causes excessive activation of immune cells and release of many proinflammatory mediators (Guo et al. 2019; Kitada et al. 2019), leading to more serious lung injury (Mowery et al. 2020). Therefore, inhibiting the production of proinflammatory mediators is of great significance.

Nicotinamide (NAM) is an adjuvant drug that is an amide compound of nicotinic acid. NAM is an important precursor of NAD and NADP in vivo (Mehmel et al. 2020). Many studies have shown that NAM is involved in anti-inflammatory and anti-aging processes (Yoshino et al. 2018). Previous studies have shown that NAM can significantly inhibit the secretion of cytokines and the chemotaxis of inflammatory cells in inflammatory skin diseases (Villeda-González 2020). Studies have also shown that NAM can decrease the number of inflammatory macrophages in skin cancer induced by chemical factors (Elhassan et al. 2019). Moreover, previous studies of the anti-inflammatory and antinociceptive activities of NAM have shown that it can effectively relieve inflammatory pain in mice, providing strong evidence for the anti-inflammatory activity of NAM (Castro-Marrero et al. 2016). Although previous studies have shown that NAM has anti-inflammatory activity, its anti-inflammatory effect on lung injury and the underlying mechanism have not been reported, so this study intends to preliminarily reveal the anti-inflammatory effect of NAM.

## Materials and methods

### Drugs and reagents

Nicotinamide (NAM, purity > 98%) and DAPI were obtained from Beyotime (Shanghai, China), dimethylsulfoxide (DMSO) was obtained from Sigma Chemical Co. (St. Louis, MO, USA), and fetal bovine serum (FBS) was produced by Biowest S.A.S. (MO, USA). Dulbecco’s modified Eagle’s medium (DMEM) for cell culture was obtained from Invitrogen-Gibco (Grand Island, NY, USA). The CCK8 kit was acquired from Saint-Bio Co. (Shanghai, China). 3,3',5,5'-Tetramethylbenzidine, resorcinol, H2O2 and HEPES were purchased from Sigma Chemical Co. (St. Louis, MO, USA). TNF-α, IL-6 and IL-1β kits were obtained from Biolegend Co. (San Diego, CA92121, USA).

Primary antibodies against AKT, p-AKT, p-ERK1/2, JNK1/2, p-JNK1/2, P38, p-P38, IκBα, p-IκBα, NF-κB-p65 and NF-κB-p-p65 were purchased from Cell Signaling Technology (CST; Danvers, USA). Primary antibodies against ERK1/2 was purchased from Proteintech (IL, USA). The primary antibody against β-tubulin was obtained from Bosterbio (CA, USA). The Alexa Fluor 488-conjugated donkey anti-rabbit IgG (H + L) highly cross-adsorbed secondary antibody, Alexa Fluor 488 conjugate was purchased from Life Technologies (Carlsbad, California, USA). HRP-conjugated anti-mouse and anti-rabbit secondary antibodies were purchased from Bosterbio.

### Animals

C57BL/6 mice (8–10 weeks old, 20–25 g in weight) were purchased from Beijing HFK Bioscience Co., Ltd. (Beijing, China). The mice were housed in certified, standard laboratory cages and administered food and water ad libitum before experimental use. All the animal care and experimental procedures were conducted in accordance with the guidelines established by the Jilin University Institutional Animal Care and Use Committee.

**Fig. 1:**
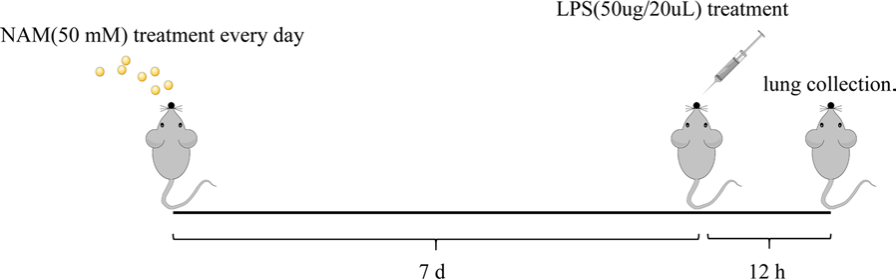
In vivo experimental procedure

The mice were randomly divided into 5 groups: the normal treatment (NT, n = 5), NAM (50 mM) treatment (n = 5), LPS (50 µg/20 µL) treatment (n = 5), LPS (50 µg/20 µL) + NAM (50 mM) treatment (n = 5), and LPS (50 µg/20 µL) + DEX (dexamethasone, 5 mg/kg) treatment (n = 5) groups. The NAM group and LPS + NAM group were provided with sterilized water containing 50 mM NAM for 7 days, and water and food were freely consumed by the mice in each group. The mice were anesthetized with ether anesthetic and inhaled LPS (50 µg/20 µL). The whole process of anesthesia and LPS treatment was very quick. Each mouse completed the operation within 5 min. After the operation, the mice were observed for 5–10 min and left after waking up and returning to normal. Then, after the inhalation of LPS for 12 h, the mice were euthanized with excessive sodium pentobarbital, and their lungs were harvested (Fig. 1).

### Protein levels of TNF-α, IL-6 and IL-1β

The protein levels of TNF-α, IL-6 and IL-1β in the lungs were determined by ELISA kits according to the manufacturer’s instructions (Gong et al. 2018).

### Myeloperoxidase (MPO) activity assay

Samples for assessing MPO activity were collected as described in a previous study (Guo et al. 2019). The lungs were harvested and weighed, and then, the samples were homogenized in 4000 μL of 0.5% hexadecyl trimethyl ammonium chloride × different lung tissue weights for 6 min (50 times per min) in a lapping instrument. After homogenization, the samples were centrifuged for 20 min at 12,000*g*/min. The supernatants were used in the subsequent MPO tests. Each sample (75 μL) and substrate (75 μL) (3,3',5,5'-tetramethylbenzidine, 3 mM, 8798 μL; resorcinol, 6 mM, 180 μL; and H_2_O_2_, 3% 2.5 μL) were added to individual wells and incubated for 3–5 min, followed by the addition of 100 μL H_2_SO_4_ (2 M) to terminate the reaction. The MPO activities of the samples were measured at OD450 with a microplate reader.

### Evaluation of histological changes

The generation of histological sections, H&E staining and histological scoring were all performed according to Lv's procedure (Liu et al. 2017). For the histological analysis of lung tissues, the mice were euthanized, and the lower lobes from the left lungs were fixed in 4% formalin, followed by dehydration with ethanol. Subsequently, 5-µm sections were cut after paraffin embedding, and the sections were stained with H&E according to a previously described protocol (Feldman and Wolfe 2014). The H&E-stained sections were observed under a light microscope to evaluate pathological changes. In addition, we used a standard assessment method to assess lung injury (Zhou et al. 2000). In detail, lung injury was scored based on edema, neutrophil infiltration, haemorrhage, bronchiole epithelial desquamation, and hyaline membrane formation, and five visual fields were observed for each slice. The observations were performed in a blinded manner. A score of 0 to 4 was used to represent the severity of injury: 0 for no damage, 1 for mild damage, 2 for moderate damage, 3 for severe damage, and 4 for very severe damage.

### Cell culture

RAW 264.7 cells were cultured in DMEM supplemented with 10% fetal bovine serum at 37 °C in a humidified incubator in 5% CO_2_. RAW 264.7 cells were cultured in 60-mm × 15-mm cell culture dishes (Life Science, Oneonta, NY, USA).

### Cell treatment

In vitro, when the cells reached approximately 70% confluence in the cell culture dishes, the DMEM supplemented with serum was replaced with serum-free DMEM. Then, 3 concentrations of NAM were added and incubated for 1 h, and LPS (1 μg/ml) was added and incubated for 3 h. Then, the cells were harvested to extract RNA in order to detect the gene expression levels of *TNF-α, IL-1β* and *IL-6*. Similar procedures were followed to detect the protein phosphorylation levels in vitro. Finally, the total proteins were extracted from the cells to detect the activation of the MAPK and Akt/NF-κB signaling pathways.

### Cell counting kit-8 assay

The effect of NAM on cell viability was determined by using a CCK-8 assay. Raw 264.7 cells were treated with LPS and NAM for 3 h. Subsequently, 10 μL CCK8 (Saint-Bio, Shanghai, China) was added to each well. After 1 h, the absorbance (OD) was measured at 450 nm with a microplate reader.

### Real-time (RT)-PCR

Total RNA was extracted from RAW 264.7 cells using TRIzol reagent. After DNase treatment, 2 μg of total RNA was reverse-transcribed into cDNA using the PrimeScriptTM RT reagent kit (TaKaRa, Kusatsu, Japan). The cDNA samples were processed for qRT-PCR as previously described. Each sample was analyzed in triplicate. The primer sequences used are presented in Table 1.

**Table 1:** Primer sequences specific for *TNF-α, IL-6* and *IL-1β*

Gene	Primer	Length (bp)
*TNF-α* (sense)	5’-ACGGCATGGATCTCAAAGAC-3’	116
*TNF-α* (anti-sense)	5’-GTGGGTGAGGAGCACGTAGT-3’	
*IL-1β* (sense)	5’-GCTGCTTCCAAACCTTTGAC-3’	121
*IL-1β* (anti-sense)	5’-AGCTTCTCCACAGCCACAAT-3’	
*IL-6* (sense)	5’-CCGGAGAGGAGACTTCACAG-3’	134
*IL-6* (anti-sense)	5’- CAGAATTGCCATTGCACAAC-3’	
*β-actin* (sense)	5’-GTCAGGTCATCACTATCGGCAAT-3’	147
*β-actin* (anti-sense)	5’-AGAGGTCTTTACGGATGTCAACGT-3’	

### Western blotting

Total proteins were harvested from lung tissues or RAW264.7 cells using RIPA lysis buffer (Beyotime, Shanghai, China; 50 mM Tris, pH 7.4, 150 mM NaCl, 1% Triton X-100, 1% sodium deoxycholate, 0.1% SDS, sodium orthovanadate, sodium fluoride, ethylenediaminetetraacetic acid, leupeptin, and 1 mM PMSF). The protein concentration was determined using a Pierce BCA Protein Assay. Western blotting was performed using standard protocols (Guo et al. 2020). The protein bands were visualized using a Beyo Enhanced Chemiluminescence reagent kit (Beyotime).

### Immunofluorescence

The lungs were fixed and processed for immunofluorescence staining as previously described (Guo et al. 2019). The primary antibody against P65 was diluted 1:150. Images of the stained sections were obtained under a fluorescence microscope.

### Statistical analysis

All the data are presented as the mean ± SD. One-way ANOVA (general linear model) was used for comparisons of more than two groups.

## Results

### NAM alleviates acute lung injury in mice.

**Fig. 2:**
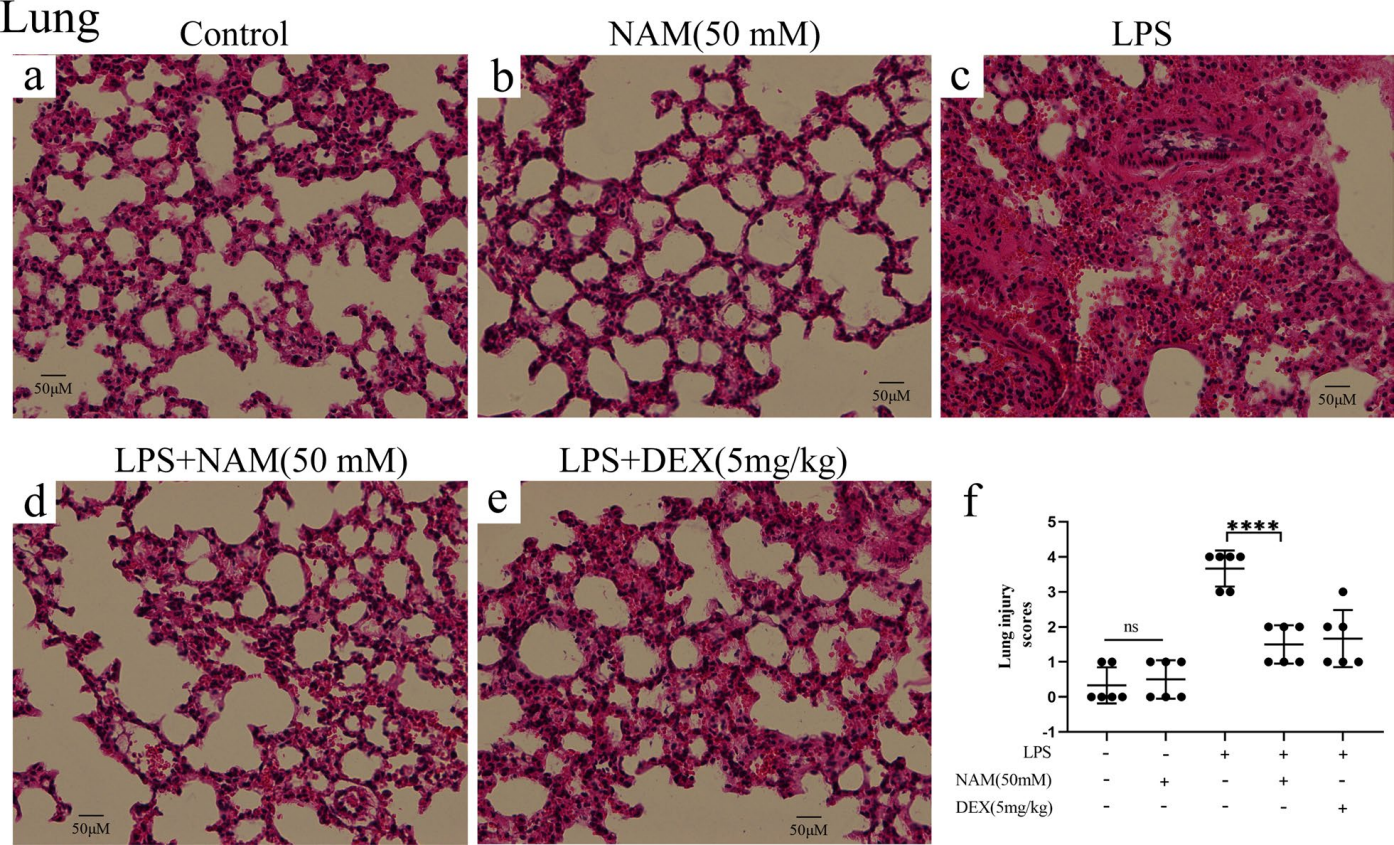
The results of H&E staining and tissue score of lung injury. Control means no treatment, NAM means NAM treatment, LPS means LPS treatment, LPS + NAM means NAM + LPS treatment, DEX means NAM + DEX treatment. **a**–**e** H&E staining of lung tissue in control, NAM, LPS, LPS + NAM and LPS + DEX groups. **f** Histological score of lung in each group. The values are presented as the mean ± SD (*p < 0.05, **p < 0.001, ***p < 0.001 and ****p < 0.0001)

Previous studies have shown that LPS-induced acute lung injury in mice is similar to that in humans. To study the effect of NAM on acute lung injury in mice, H&E staining was carried out. We found that the alveolar wall of the LPS group was thickened, with obvious edema and inflammatory cell infiltration. Moreover, the numbers of neutrophils in the lungs of the LPS group were significantly increased, which suggested that LPS caused lung injury. However, the results of the NAM treatment group were consistent with those of the dexamethasone (DEX) group. Both NAM and DEX could significantly reduce the number of neutrophils in the lungs and alleviate lung injury. The lung injury scores were consistent with the H&E staining results (Fig. 2). These results suggested that NAM can significantly improve LPS-induced acute lung injury.

### NAM reduces the secretion of proinflammatory mediators in the lung

**Fig. 3:**
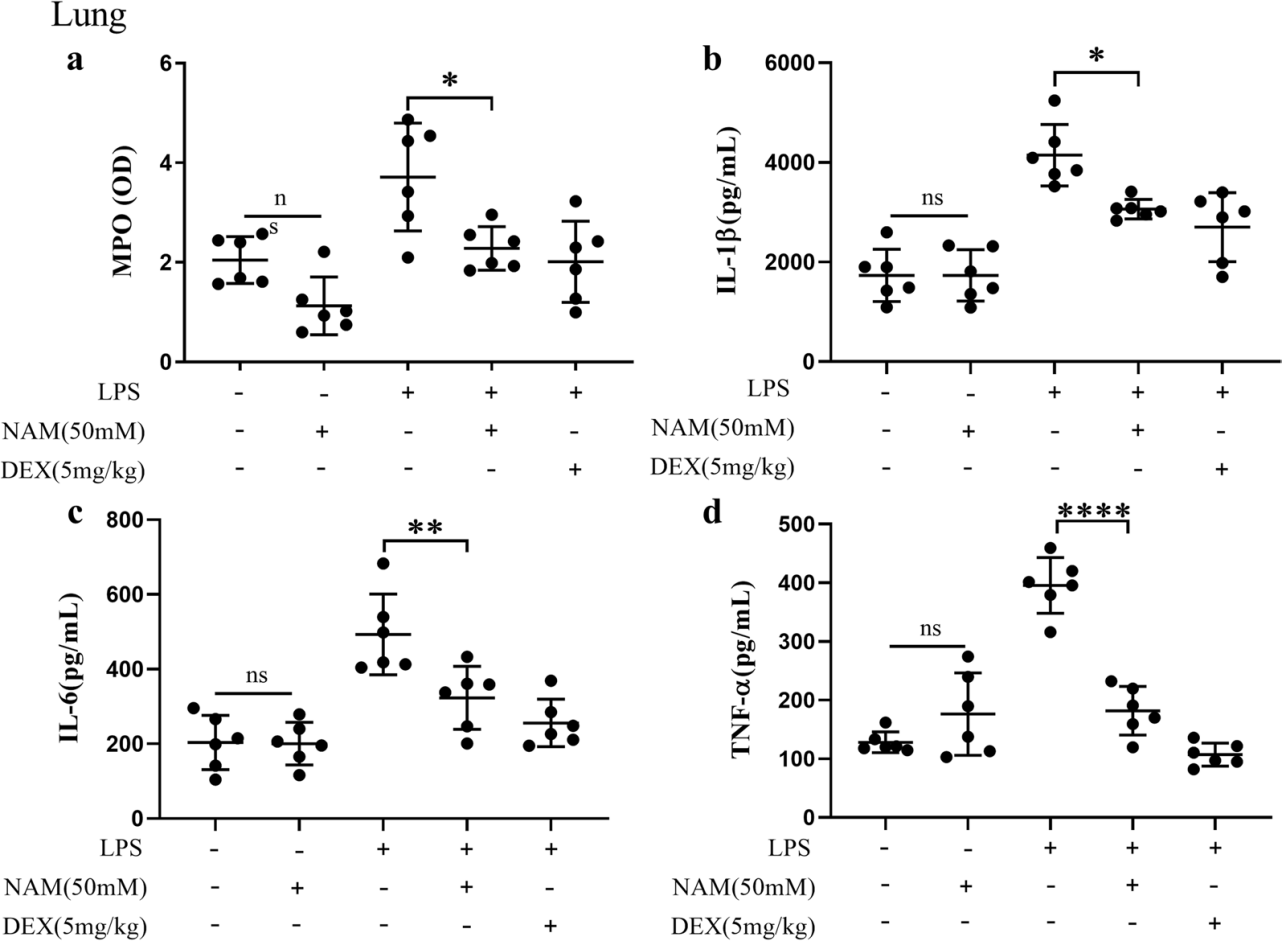
The expression of MPO and pro-inflammatory mediators in lung. **a** MPO activity test results. **b**–**d** The protein levels of IL-6, IL-1β and TNF-α. The values are presented as the mean ± SD (*p < 0.05, **p < 0.001, ***p < 0.001 and ****p < 0.0001)

Many studies have shown that a large number of proinflammatory mediators can lead to acute lung injury, which can seriously affect alveolar function and patient health. In this study, we found that the MPO activity in the LPS group was significantly increased, while the MPO activity in the control group and NAM treatment group was significantly decreased. Then, we detected the expression of TNF-α, IL-1β and IL-6 and found that NAM treatment alone did not lead to an increase in the expression of these proinflammatory mediators. LPS treatment significantly promoted the expression of TNF-α, IL-1β and IL-6, and NAM treatment significantly reduced the expression of TNF-α, IL-1β and IL-6. This result is also consistent with the trend observed in the DEX treatment group (Fig. 3). Then we used macrophages in alveolar lavage fluid to study. The results showed that the gene levels of *TNF-α, IL-1β* and *IL-6* in alveolar macrophages was significantly down regulated after the addition of NAM (Additional file 1: S2a–c). These results suggested that NAM may alleviate acute lung injury by inhibiting the expression of proinflammatory mediators.

### NAM suppresses the MAPK and AKT/NF-κB signaling pathways in the lung

**Fig. 4:**
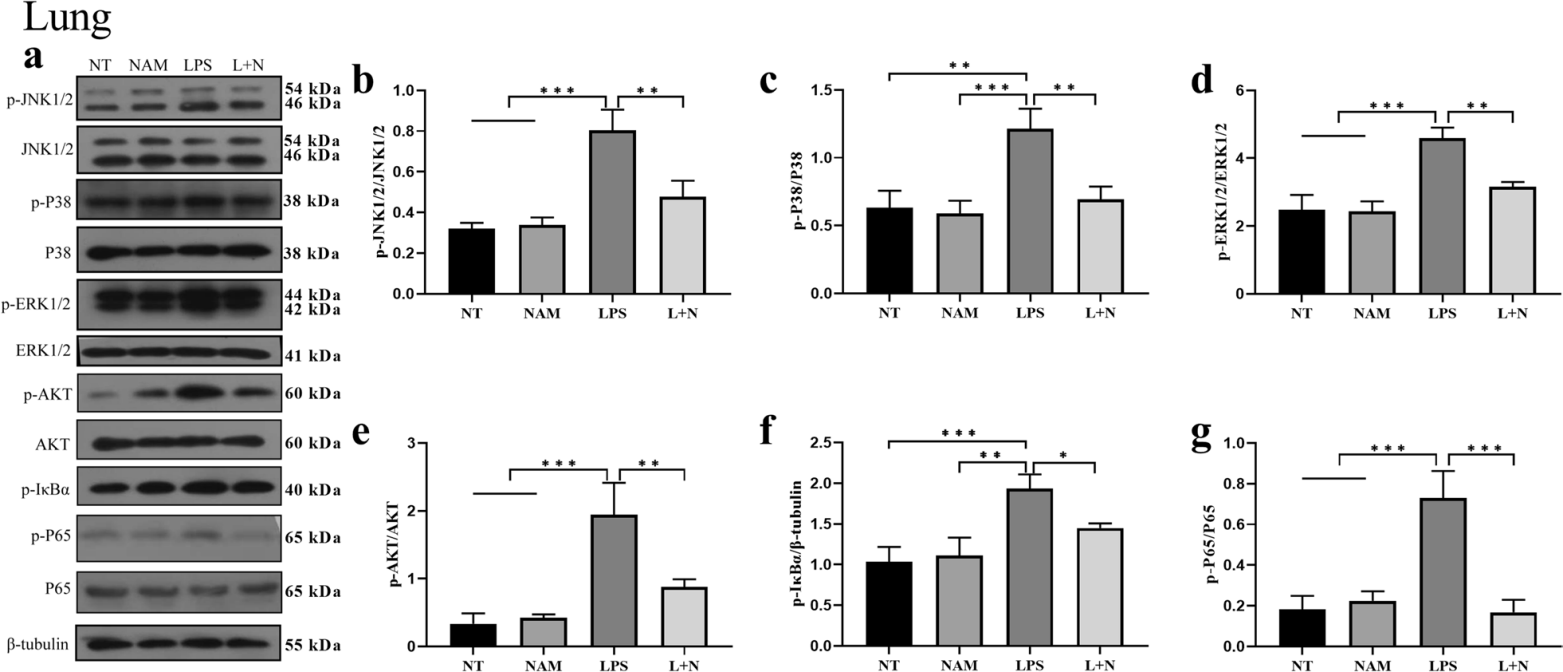
The effect of NAM on MAPK and NF-κB signaling pathway in lung. **a**–**g** Lung tissues from different experimental groups were obtained 12 h after LPS administration and total protein extracted. The phosphorylation levels of ERK, JNK, P38, AKT, IκBα and P65 were determined via western blot and quantitated via densitometry using β-tubulin as an internal control. The values are presented as the mean ± SD (*p < 0.05, **p < 0.001, ***p < 0.001 and ****p < 0.0001)

Some studies have shown that the MAPK and AKT/NF-κB signaling pathways are significantly activated during acute lung injury, and the activation of these pathways promotes the secretion of inflammatory mediators. In this study, we found that LPS could significantly promote the phosphorylation of ERK, JNK and P38 in the lung. Then, we assessed the AKT/NF-κB signaling pathway and found that the levels of phosphorylated Akt, IκBα and p65 were also significantly increased. This suggested that LPS may enhance lung injury by activating these signaling pathways. Then, we pretreated the mice with NAM prior to inducing lung injury with LPS. We found that NAM significantly inhibited the phosphorylation of ERK, JNK and p38. Then, we detected the levels of phosphorylated Akt, IκBα and p65 and found that NAM inhibited the phosphorylation of these proteins (Fig. 4). At the same time, we also detected the phosphorylation level of P65, a key pro-inflammatory pathway protein, in alveolar macrophages. The results showed that NAM could significantly inhibit the phosphorylation of P65 (Additional file 1: S2d, e).The results showed that NAM may alleviate acute lung injury by inhibiting the MAPK and AKT/NF-κB signaling pathways.

### Effect of NAM on RAW264.7 cell viability

**Fig. 5:**
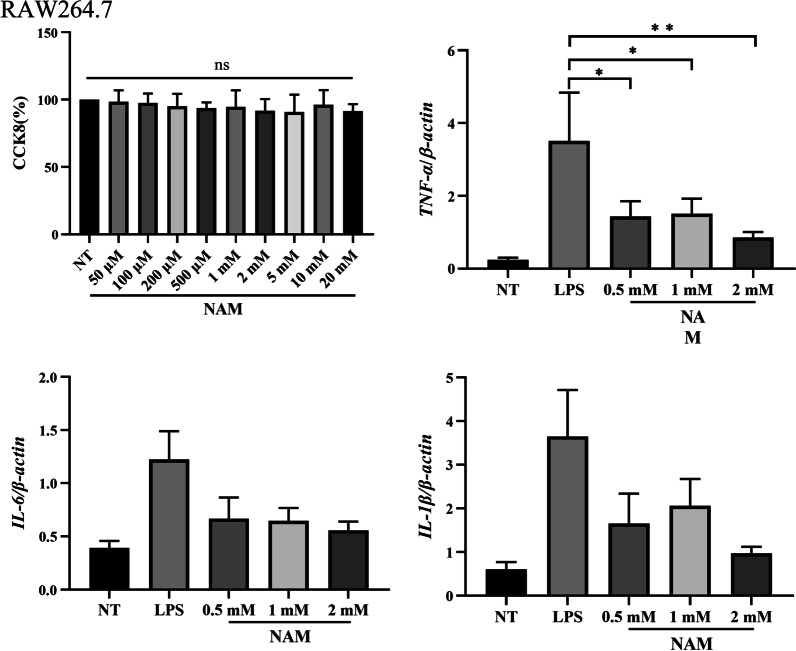
The effect of NAM on cell viability and pro-inflammatory related genes in RAW264.7. **a** The effect of NAM on cell viability. The effect of NAM on cell activity was detected by CCK. The concentrations of NAM were 50 μM, 100 μM, 200 μM, 500 μM, 1 mM, 2 mM, 5 mM, 10 mM and 20 mM respectively. **b**–**d** The gene levels of *IL-6, IL-1β* and *TNF-α* were detected using qRT-PCR in raw264.7 and normalized to that of *β-actin*. The values are presented as the mean ± SD (*p < 0.05, **p < 0.001, ***p < 0.001 and ****p < 0.0001)

In this experiment, different concentrations of NAM were added to RAW264.7 cell cultures. The results showed that the activity of RAW264.7 cells was not affected by NAM at concentrations below 20 mM (Fig. 5a).

### NAM can inhibit the gene expression of *IL-6, TNF-α *and *IL-1β* in RAW264.7 cells and primary macrophages

Previous studies have shown that acute lung injury is mainly caused by proinflammatory mediators secreted by macrophages. Therefore, inhibiting the secretion of proinflammatory mediators by macrophages is of great significance for alleviating acute lung injury. We found that LPS could significantly promote the expression of *IL-6, TNF-α* and *IL-1β* in RAW264.7 cells, but the gene expression of *IL-6, TNF-α* and *IL-1β* was significantly inhibited by the addition of NAM (Fig. 5b–d). Then we isolated primary macrophages and carried out experiments. The results showed that NAM could also significantly inhibit the expression of *IL-6, TNF-α* and *IL-1β* in primary macrophages (Additional file 1: S1). This result suggested that NAM may reduce lung injury by inhibiting the secretion of proinflammatory mediators by RAW264.7 cells.

### NAM inhibits the MAPK and AKT/NF-κB signaling pathways in RAW264.7 cells

**Fig. 6:**
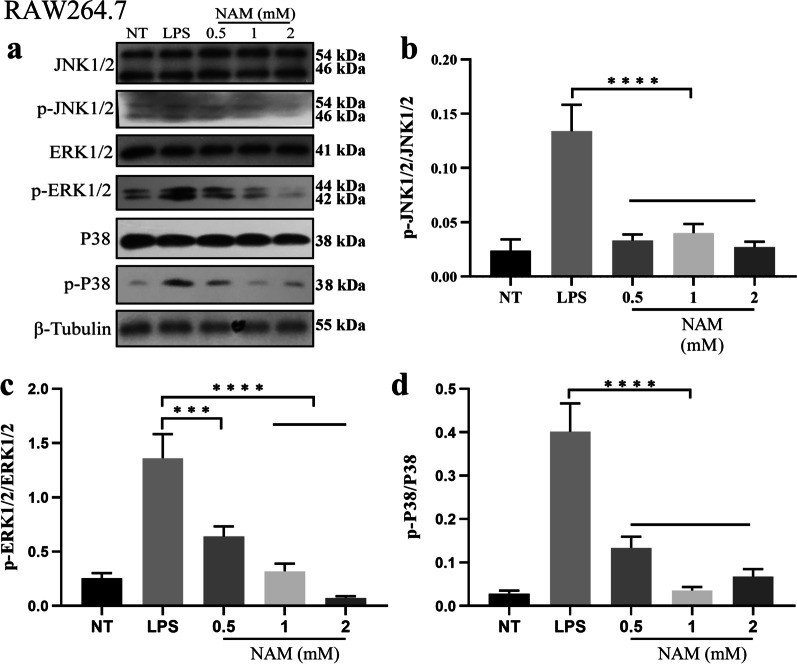
Effect of NAM on MAPK signaling pathway in RAW264.7. The experiment was divided into 5 groups: NT, LPS, LPS + 0.5 mM, LPS + 1 mM and LPS + 2 mM. **a**–**d** NAM was added 1 h before LPS was added, and the total protein of the cells was collected after LPS treatment for 3 h. The phosphorylation of ERK, JNK and P38 were detected by western blot. The values are presented as the mean ± SD (*p < 0.05, **p < 0.001, ***p < 0.001 and ****p < 0.0001)

**Fig. 7:**
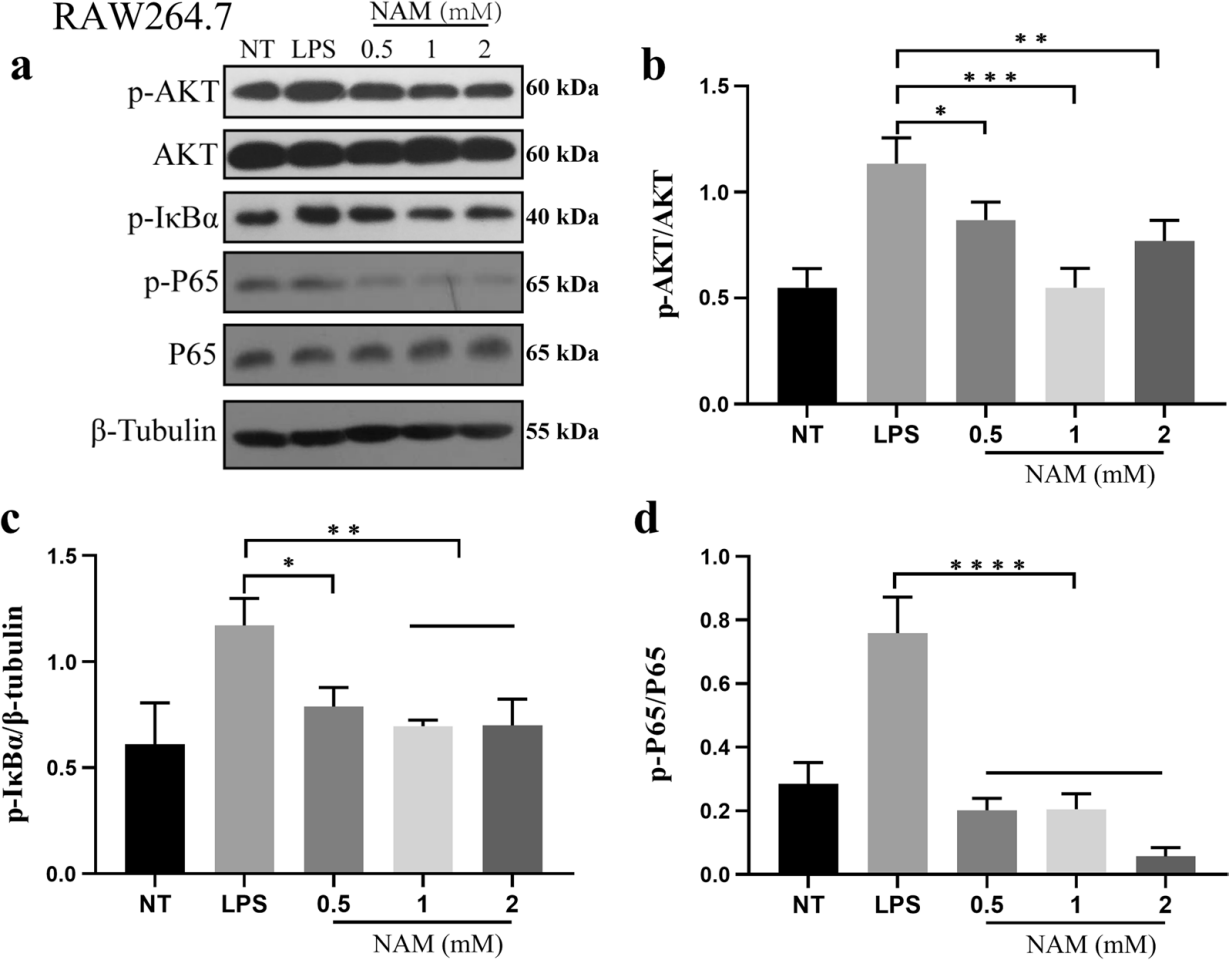
Effect of NAM on AKT/NF-κB signaling pathway in RAW264.7. The experiment was divided into 5 groups: NT, LPS, LPS + 0.5 mM, LPS + 1 mM and LPS + 2 mM. **a**–**d** NAM was added 1 h before LPS was added, and the total protein of the cells was collected after LPS treatment for 3 h. The phosphorylation of AKT, IκBα and P65 were detected by western blot. The values are presented as the mean ± SD (*p < 0.05, **p < 0.001, ***p < 0.001 and ****p < 0.0001)

Recent studies have shown that activation of the MAPK and AKT/NF-κB signaling pathways can activate macrophages, leading to the release of a large number of proinflammatory mediators and eventually causing acute lung injury. To explore the mechanism underlying macrophage activation, we examined the MAPK and AKT/NF-κB signaling pathways and found that LPS could significantly activate the MAPK and AKT/NF-κB signaling pathways. Then, we added NAM to LPS-stimulated RAW264.7 cells. The results showed that NAM significantly inhibited the levels of phosphorylated ERK, JNK, P38, AKT, IκBα and P65 (Figs. 6–7). These results suggested that NAM can inhibit the activation of the MAPK and AKT/NF-κB signaling pathways.

### NAM prevents the nuclear translocation of p-P65

**Fig. 8:**
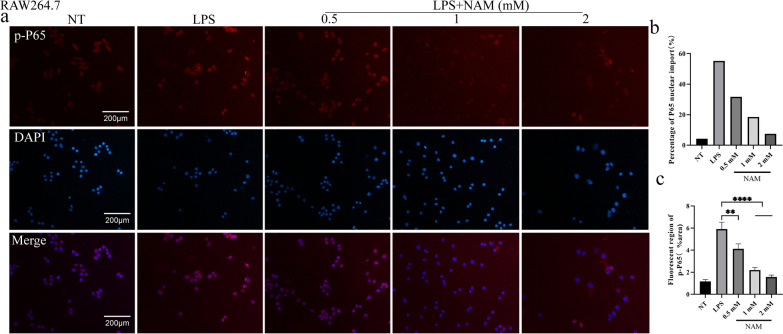
The fluorescence intensity and nuclear import of p-P65 in RAW264.7 cells. The nucleus is blue and p-P65 is red. **a** Fluorescence intensity and nuclear import of p-P65 after different treatments. **b** Percentage of p-P65 nuclear import

P65 can significantly promote the expression of downstream proinflammatory mediators. We found that LPS could significantly promote the translocation of P65 into the nucleus. However, our study showed that NAM significantly inhibited the nuclear translocation of p-P65, which indicates that NAM can inhibit the expression of proinflammatory genes by inhibiting the nuclear translocation of p-P65 (Fig. 8).

## Discussion

The lung injury caused by bacterial pneumonia is mainly driven by pathogen-associated molecular patterns (Tolle and Standiford 2013). LPS, an important pathogen-related molecule, can cause severe inflammatory reactions in the lung (Nova et al. 2019). Studies have shown that LPS can cause alveolar macrophages to release large amounts of IL-6, IL-1β and TNF-α, which cause acute lung damage (Ehrentraut et al. 2019). IL-6 is an important inflammatory factor (Tanaka et al. 2014) whose levels sharply increase in the process of bacterial infection and seriously affect the lung microenvironment (Wang et al. 2018). Previous studies have shown that NAM can significantly inhibit the pulmonary infiltration of neutrophils in ventilator-induced lung injury, thus playing an anti-inflammatory role (Jones 2015). Although the mechanism underlying ventilator-induced lung injury may be different from that underlying LPS-induced lung injury, both models lead to the secretion of a large number of proinflammatory mediators, and NAM can significantly inhibit the release of these proinflammatory mediators (Jones 2015). Our study found that NAM could effectively reduce the accumulation of IL-6 in the lung, and the cell experiments also showed that NAM could inhibit the expression of IL-6 by macrophages. IL-1β can activate Toll-like receptors in macrophages (Xu et al. 2020), thus exacerbating the inflammatory response (Gabay et al. 2010). Our research showed that NAM could reduce the expression of IL-1β in the lung, and we also found that NAM could inhibit the gene expression of IL-1β in macrophages. TNF-α is mainly secreted by macrophages and monocytes (Parameswaran and Patial 2010). Studies have shown that TNF-α is involved in the occurrence and development of inflammation (Bradley 2008; Hayden and Ghosh 2014). Our study showed that LPS could significantly increase the level of TNF-α, while NAM could inhibit the release of TNF-α. We found that NAM could inhibit the gene expression of IL-1β in vitro. The studies described above show that NAM can alleviate the inflammatory response in the lung by inhibiting the release of inflammatory mediators, such as IL-6, IL-1β and TNF-α.

Many studies have shown that LPS-induced acute lung inflammation can lead to severe lung injury (Lee et al. 2018), and some natural products or compounds can alleviate acute lung injury and improve the success of lung injury treatment (St Croix et al. 2005). To verify whether NAM can reduce lung injury in mice, H&E staining was carried out. The results showed that the alveolar walls of the mice in the LPS group were significantly thickened, and significant infiltration of neutrophils was observed. However, the lung injury of mice was significantly improved after treatment with NAM. These results suggest that NAM can significantly reduce lung injury.

Although we found that NAM can significantly alleviate inflammatory lung injury, the specific mechanism is not clear. Therefore, in follow-up experiments, we studied the anti-inflammatory mechanism of NAM. Many studies have shown that the AKT/NF-κB and MAPK signaling pathways play important roles in the inflammatory response (Yeung et al. 2018; Sun et al. 2019; Guo et al. 2015). LPS can promote the expression of downstream proinflammatory mediators by activating the AKT/NF-κB signaling pathway (Lee et al. 2011; Zhang et al. 2011; He et al. 2019). Inhibition of the AKT/NF-κB signaling pathway can significantly alleviate the inflammatory response (Tang et al. 2018). Therefore, we detected AKT/NF-κB signaling pathway-related proteins after treatment with NAM and found that NAM significantly inhibited the phosphorylation of AKT and IκBα, thereby reducing the phosphorylation of P65. We found that NAM could also significantly inhibit the phosphorylation of ERK1/2, P38 and JNK1/2, thereby reducing the activation of the MAPK signaling pathway, ultimately inhibiting the release of proinflammatory mediators and reducing the inflammatory response.

## Conclusion

**Fig. 9:**
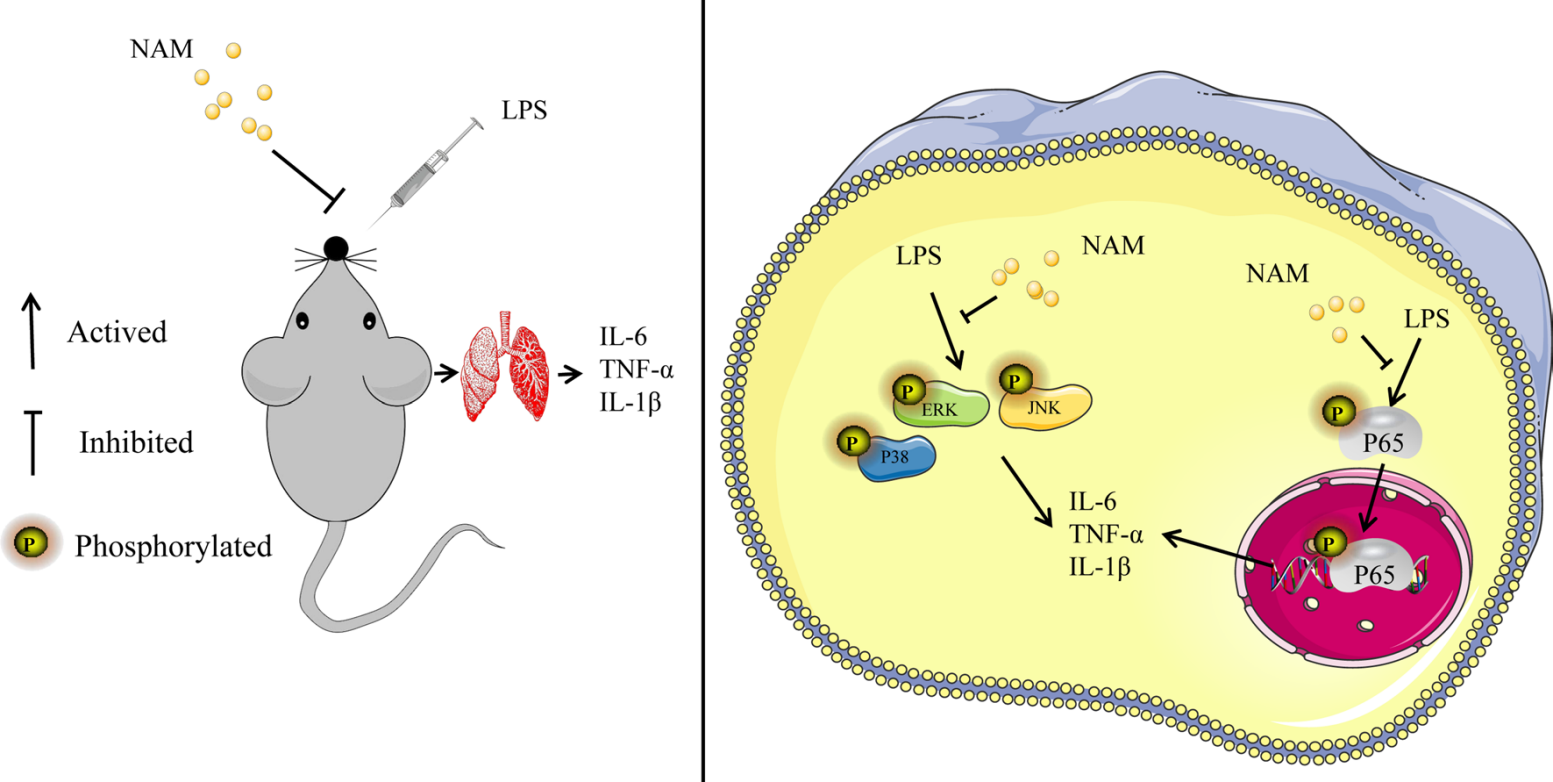
Mechanism of NAM alleviating LPS induced lung injury

In vitro, we found that NAM can significantly inhibit the expression of proinflammatory mediators by inhibiting the MAPK and Akt/NF-κB signaling pathways. In in vivo experiments, we used RAW264.7 cells as the research object. The results also showed that NAM can significantly reduce the gene expression of *IL-6, IL-1β* and *TNF-α*, and this process is also closely related to the MAPK and Akt/NF-κB signaling pathways. Therefore, NAM has potential prospects in the treatment of lung injury (Fig. 9).

## Supplementary Information

**Additional file 1: Fig. S1. **Effect of NAM on *IL-6, TNF-α *and* IL-1β* in primary macrophages. Primary mouse macrophages were isolated and treated with NAM and LPS. The treatment method was the same as that in RAW264.7 cells. (a-c) The gene levels of* IL-6, TNF-α *and* IL-1β *were detected using qRT-PCR in raw264.7 and normalized to that of *β-actin. *The values are presented as the mean ± SD (*p < 0.05, **p < 0.001, ***p < 0.001 and ****p < 0.0001). **Fig. S2.** Effect of NAM on alveolar macrophages. The alveolar lavage fluid of mice was collected to isolate and screen the macrophages in the lungs of mice. Then RNA and protein from macrophages were extracted for test. (a–c) Effect of NAM on gene levels of IL-6、TNF-α and IL-1β in pulmonary macrophages. (d–e) Inhibitory effect of NAM on p-P65 in pulmonary macrophages. The values are presented as the mean ± SD (*p < 0.05, **p < 0.001, ***p < 0.001 and ****p < 0.0001).

## Data Availability

The datasets used or analysed during the current study are available from the corresponding author on reasonable request.
