# Supplementary material for: The mechanism of nicotinamide on reducing acute lung injury by inhibiting MAPK and NF-κB signal pathway
Source: Mol Med. 2021 Sep 20;27:115. doi: 10.1186/s10020-021-00376-2 (PMC8451170; doi:10.1186/s10020-021-00376-2)
Supplement: Supplementary file 1 — Additional file 1: Fig. S1. Effect of NAM on IL-6, TNF-α and IL-1β in primary macrophages. Primary mouse macrophages were isolated and treated with NAM and LPS. The treatment method was the same as that in RAW264.7 cells. (a-c) The gene levels of IL-6, TNF-α and IL-1β were detected using qRT-PCR in raw264.7 and normalized to that of β-actin. The values are presented as the mean ± SD (*p < 0.05, **p < 0.001, ***p < 0.001 and ****p < 0.0001). Fig. S2. Effect of NAM on alveolar macrophages. The alveolar lavage fluid of mice was collected to isolate and screen the macrophages in the lungs of mice. Then RNA and protein from macrophages were extracted for test. (a–c) Effect of NAM on gene levels of IL-6、TNF-α and IL-1β in pulmonary macrophages. (d–e) Inhibitory effect of NAM on p-P65 in pulmonary macrophages. The values are presented as the mean ± SD (*p < 0.05, **p < 0.001, ***p < 0.001 and ****p < 0.0001). [file 10020_2021_376_MOESM1_ESM.docx]

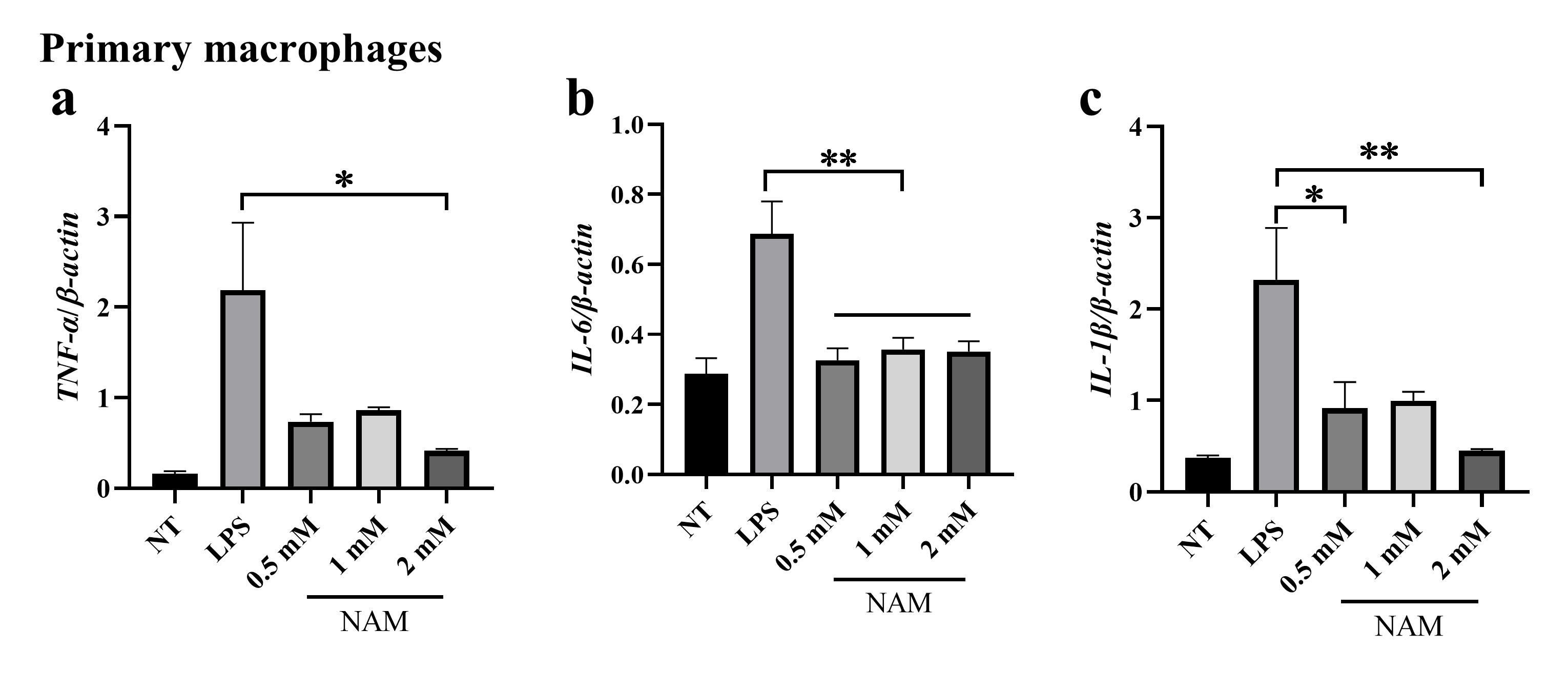


S1. Effect of NAM on *IL-6, TNF-α* and *IL-1β* in primary macrophages. Primary mouse macrophages were isolated and treated with NAM and LPS. The treatment method was the same as that in RAW264.7 cells. (a-c) The gene levels of *IL-6, TNF-α* and *IL-1β* were detected using qRT-PCR in raw264.7 and normalized to that of *β-actin.* The values are presented as the mean ± SD (**p<*0.05*, **p<*0.001*, ***p<*0.001 and *****p<*0.0001)*.*


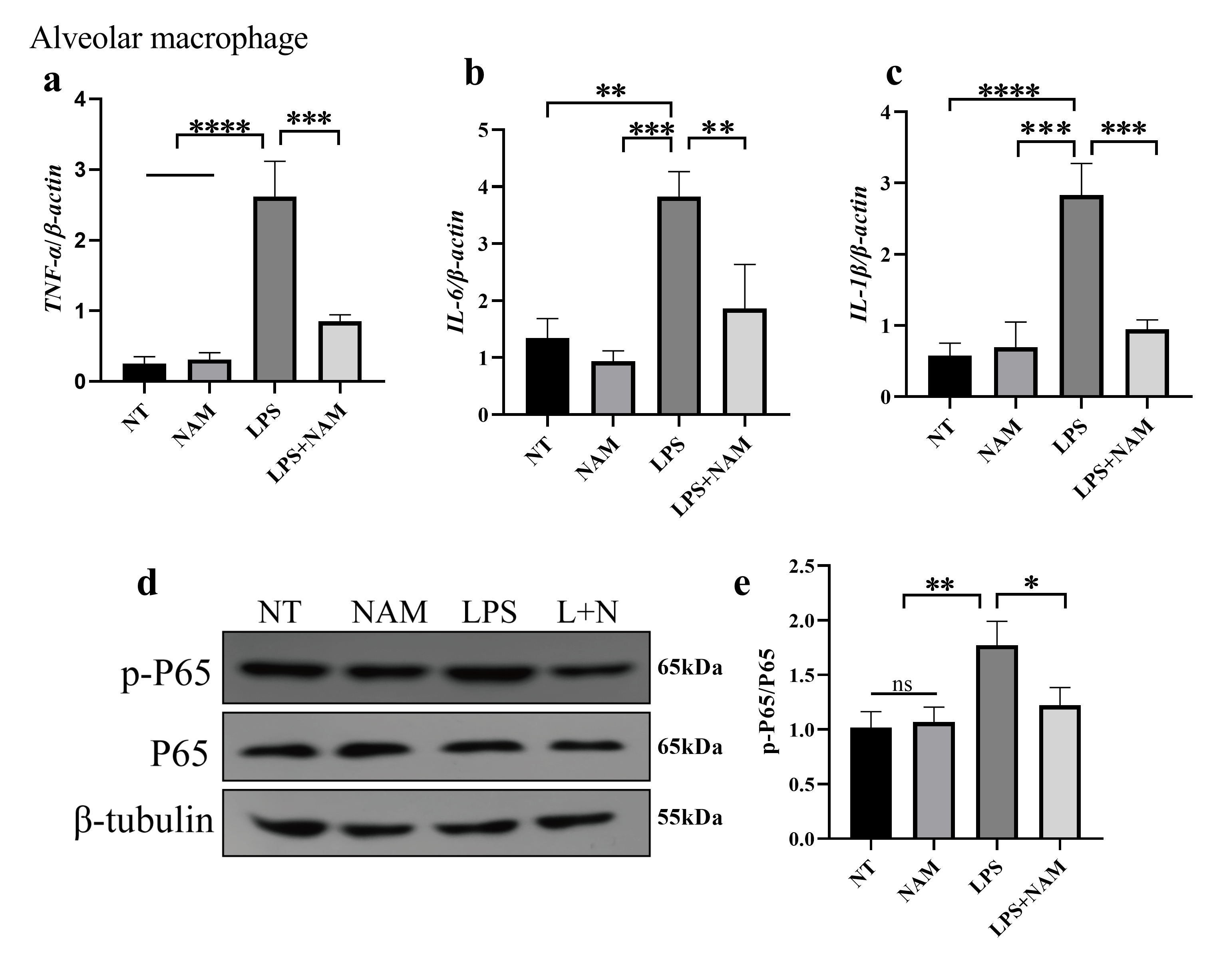


Fig. S2. Effect of NAM on alveolar macrophages. The alveolar lavage fluid of mice was collected to isolate and screen the macrophages in the lungs of mice. Then RNA and protein from macrophages were extracted for test. (a-c) Effect of NAM on gene levels of IL-6、TNF-α and IL-1β in pulmonary macrophages. (d-e) Inhibitory effect of NAM on p-P65 in pulmonary macrophages. The values are presented as the mean ± SD (**p*<0.05, ***p*<0.001, ****p*<0.001 and *****p*<0.0001).
